# A 2.5‐year snapshot of Mendelian discovery

**DOI:** 10.1002/mgg3.221

**Published:** 2016-04-03

**Authors:** Benjamin D. Solomon, Teresa Lee, Anh‐Dao Nguyen, Tyra G. Wolfsberg

**Affiliations:** ^1^ Division of Medical GenomicsInova Translational Medicine InstituteInova Health SystemsFalls ChurchVirginia22042; ^2^ Department of PediatricsInova Children's HospitalInova Health Systems Falls ChurchVirginia22042; ^3^ Department of PediatricsVirginia Commonwealth University School of MedicineRichmondVirginia23298; ^4^ Computational and Statistical Genomics BranchNational Human Genome Research InstituteBethesdaMaryland20892

## Introduction

New sequencing technologies are becoming increasingly available in a variety of contexts. These techniques include whole‐exome (we refer to this as “exome” sequencing) and whole‐genome sequencing (Biesecker and Green 2014). These types of genomic sequencing have impacted both research and clinical practice. Genomic sequencing has led to the discovery of novel genetic etiologies of disease and has also improved the ability to diagnose patients with subtle or atypical presentations of genetic conditions (especially those affected by relatively rare disorders) by allowing simultaneous interrogation of many loci (Boycott et al. 2013; Yang et al. 2013, 2014; Taylor et al. 2015).

To quantify genetic progress and the impact of these technologies in understanding the causes of Mendelian disorders, we analyzed methods of discovery in the last ~2.5 years.

## Materials and Methods

We reviewed all newly described Mendelian disease genes in the ~2.5‐year period (April 30, 2013–November 30, 2015) following the initial public dissemination of the Clinical Genomic Database (CGD) (Solomon et al. 2013), a freely available web‐based resource that focuses on the clinical sequelae and management of genetic disorders (the CGD, which is regularly updated to keep pace with genetic knowledge, is available at: http://research.nhgri.nih.gov/CGD/). Regarding relevant literature, as there is frequently a gap between manuscript acceptance, electronic, and final publication, we attempted to include only those genes/conditions in the ~2.5 year period that were published (available in PubMed) and subsequently included in both the CGD and Online Mendelian Inheritance in Man (OMIM, available at http://www.omim.org) within that ~2.5 year interval. We recognize this is imperfect for several reasons. These reasons include the lag between publication and incorporation in these databases, and the fact that the databases do not completely capture the literature and are themselves being continually being refined and updated. However, with these caveats in terms of potential inaccuracies, the trends that we reveal are overall interesting.

For each article, we determined how the discovery was made (e.g., through whole‐genome sequencing alone, homozygosity mapping and exome sequencing, candidate gene studies, or a combination of possibilities). If the discovery involved findings through a previous publication on the same families or condition (such as linkage analysis implicating specific loci), we included that previous method as part of the discovery process. In the example given, this would be considered “exome + linkage.” We also determined if the discovery was made through analysis of a single individual; a single family; or a single individual or family followed by the identification of additional mutation‐positive individuals through studies of a larger cohort. Finally, we investigated whether additional bench‐based studies, aimed at providing understanding and evidence beyond clinical and bioinformatics results, were included in the investigation. We did not include newly reported conditions allelic to previously described conditions. Regarding this latter exclusion criterion, though many genes have clinically distinct allelic disorders, we wished to be maximally conservative in order to avoid any controversy arising from similar disorders that may represent the spectrum of a single disease entity.

## Results

**Figure 1:**
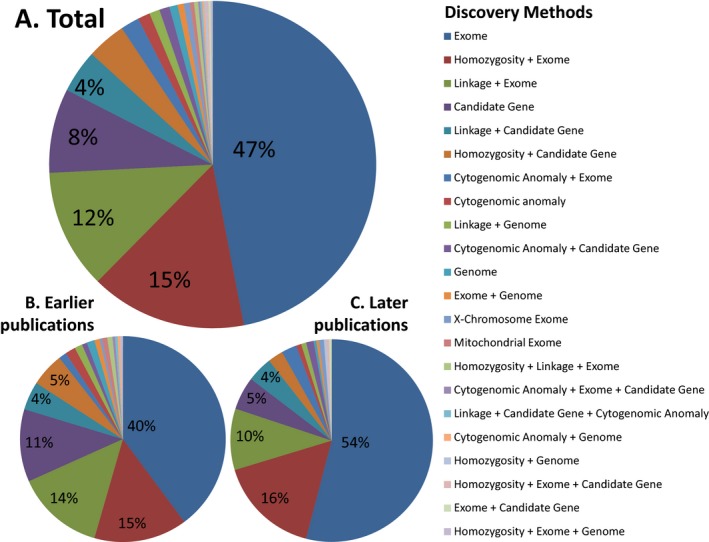
Discovery methods related to the 445 genes (representing 492 studies) newly identified as involved in human Mendelian disease within the selected ~2.5‐year period. The top chart (A) shows the overall breakdown of methods within the 2.5‐year period; (B) shows the rough first chronological half of publications (sorted by PubMed IDs), while (C) shows the rough second chronological half. There was significantly more use of genomic sequencing, both alone and in conjunction with other techniques, in the second half of the period.

For newly discovered genetic causes, 445 new genes were identified in the 2.5‐year period in the defined intervals (see Fig. 1 and Table S1 for details). A number of genes were identified by apparently independent studies with separate but simultaneous publications. Methods from each such independent study were counted separately here, such that 492 individual studies are represented. Of the 492 studies, 394 (80%) used some type of genomic sequencing (including exome or genome sequencing but not X‐chromosome exome or mitochondrial exome). Three hundred and seventy‐nine (77%) used exome sequencing, 11 (2%) used genome sequencing, and four (1%) used both exome and genome sequencing in the same study. Two hundred and thirty‐one (47%) used only exome sequencing (without other investigations such as homozygosity mapping) for gene identification; 238 (48%) used only exome and/or genome sequencing. Another 156 (32%) used exome or genome sequencing in addition to other techniques, such as homozygosity mapping. Forty‐one (8%) used a traditional candidate gene approach without other methods, though 46 (10%) used a candidate gene approach with methods other than exome or genome sequencing. The remaining 11 (2%) used other methods, such as cytogenomic methods alone, X‐chromosome exome sequencing, or mitochondrial sequencing (see Table S1).

Eighteen (4%) of the overall studies investigated a single patient only; 114 (23%) studied multiple members of a single family; 93 (19%) were initially done on a single patient or family but then investigated a larger cohort based on these initial findings and identified and reported additional mutation‐positive individuals. The remaining 267 (54%) were reported as studying multiple patients or families simultaneously.

We would predict that over time, the use of genomic sequencing methods will eclipse other methods of identifying disease genes. To detect if there has been a shift in methods, even over the short ~2.5 year period of this study, we sorted the 492 publications by PubMed identification number (PMID) and split the publications into two groups. We recognize that the PMID identifiers do not precisely capture chronology. Nevertheless, we note that studies in the second group, which represents manuscripts published more recently, made significantly more use of genomic sequencing overall (210 vs. 184 studies, *P* = 0.0046 by Fisher's exact test) and genomic sequencing alone (135 vs. 103 studies, *P* = 0.0051 by Fisher's exact test) than did those in the first.

Of the 492 studies, 359 (73%) included some type of laboratory‐based assay in addition to standard clinical work‐up and genomic sequencing and analysis. However, we intentionally did not further analyze the type of cellular or functional analyses that were done as part of the paper – we viewed this assignment as difficult and potentially unhelpful to interpret, as this depended on the availability of previous knowledge about a particular gene's function (e.g., through an animal model), the specific mutation in question (e.g., a truncating vs. missense mutation), may have been aimed at understanding the biological implications of the genetic pathways involved rather than or in addition to the question of mutation pathogenicity, and because so many different possible methods might have been used (MacArthur et al. 2014).

## Discussion

Our analyses show that exome sequencing currently accounts for the vast majority of causal gene discovery. Almost half of all the discoveries investigated here occurred through exome sequencing alone. This is anticipated to shift to genome sequencing as affordability and accuracy of sequencing continue to improve (Hayden 2014). Additionally, our analyses show a statistically significant increase in the use of exome or genome sequencing even within the short time period analyzed. Again, though we admit to potential inaccuracies as the background databases shift, we feel that the trends described here are illustrative.

In conclusion, new sequencing technologies are resulting in a dramatic change in the discovery of the causes of disease. These discoveries can be quickly translated into clinical care – patients affected with many conditions now have a better chance of explanations based on genetic testing. Further, finding the molecular etiology of a condition may then in turn yield more tailored and overall better management (Solomon et al. 2013; Soden et al. 2014; Khromykh and Solomon 2015; Solomon 2015; Willig et al. 2015). It will be interesting to perform similar analyses in the future as new techniques emerge and as the body of knowledge of the causes of human disease continues to grow and evolve.

## Conflict of Interest

None of the authors have disclosures or conflicts of interest.

## Supporting information


**Table S1.** Spreadsheet of genes, conditions, and articles included in this analysis.
